# Vaccination Officers

**Published:** 1896-03-21

**Authors:** 


### EDITOR'S LETTER-BOX.

### VACCINATION OFFICERS.

Mr. Shirley Fussell, Secretary to the National Poor Law Officers' Association, writes: The National Association of Poor Law Officers of England and Wales desire to draw public attention to the anomalous position in which vaccination officers are placed through the delay in the report of the Royal Commission on Vaccination. This Commission was appointed on May 29th, 1889, and the warrant of appointment concludes as follows: "And our further will and pleasure is that you do, with as little delay as possible, report to us under your hands and seals, or under the hands and seals of any five or more of you, your opinion upon the several matters herein submitted for your consideration." In March, 1896, however, the report is not forthcoming, and vaccination officers, together with the general public, have to rest content with the assurance that "the Commission will report in due course." Boards of Guardians are constantly calling attention to the inconvenience to which they are put by this long delay, but to the vaccination officers, who are paid by results, the delay is of vital importance. The Guardians and the Local Government Board have been approached upon the subject of the vaccination officers' diminishing incomes, but have felt themselves precluded from dealing with the matter pending the report of the Royal Commission, and the vaccination officers feel that they have no alternative than to seek the aid of the Press to bring their grievances before the public.

